# Chronic, Stromal Foreign Body of Presumed Human Origin, following Corneal Abrasion

**DOI:** 10.1155/2019/9607282

**Published:** 2019-03-19

**Authors:** Alexander K. Soon, Rookaya Mather

**Affiliations:** ^1^Department of Ophthalmology, University of Western Ontario, London, ON, Canada; ^2^The Ivey Eye Institute, London, ON, Canada

## Abstract

A 30-year-old woman presented with left eye pain and photophobia for over eight weeks. A hair was found to be embedded obliquely within the corneal stroma with overlying reepithelialization. The foreign body had been quiescent for over four years prior to any ocular symptoms. Successful removal of the hair was performed using local anesthetic, jeweler's forceps, a microblade, and a thirty-gauge needle with good visual outcome. We report an interesting case of an acute exacerbation of a previously quiescent, chronic corneal foreign body secondary to what we presume to be human hair following trauma, with only three other cases in the English literature.

## 1. Introduction

There have been plentiful reports of animal hair, such as tarantulas and caterpillars, causing simple conjunctivitis, keratouveitis, panuveitis, chorioretinitis, and ophthalmia nodosa following trauma [1, 2]. However, reports of human hair embedded in the cornea are rare [3, 4] We report the first case of a corneal foreign body consisting of a presumed human hair follicle following a corneal abrasion in Canada.

## 2. Case

A 30-year-old woman was referred by a local optometrist to the Emergency Eye Clinic at the Ivey Eye Institute with a ten-week history of left eye foreign-body sensation, pain, and photophobia. There was no recent traumatic event that she could recall. She was placed on seven-day course of prednisolone drops with resolution of her symptoms for approximately four weeks. However, her symptoms recurred and she was again treated with another short course of mild steroid drops with minor alleviation of her symptoms. Two days after this, her symptoms returned and, thus, she was referred to ophthalmology for a concern of a chronic foreign body of the left cornea.

**Figure 1:**
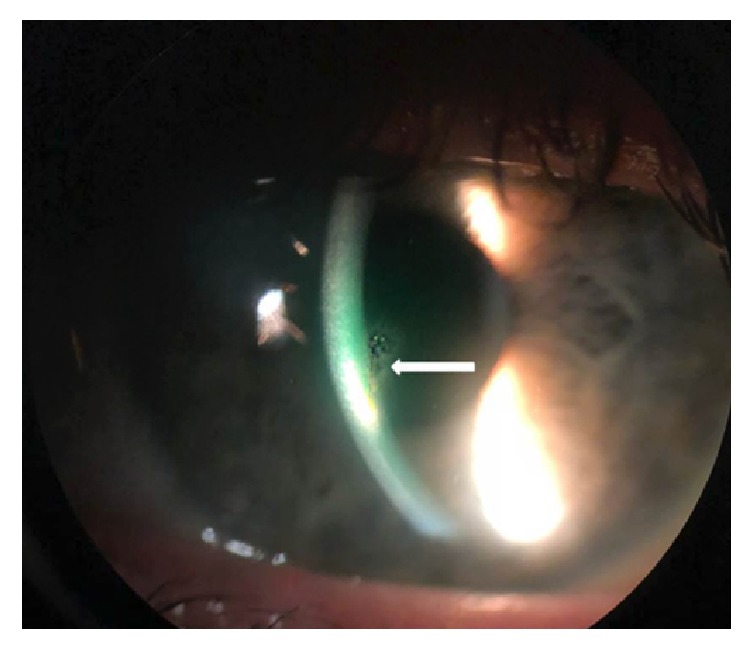
Slit lamp photograph of the left eye demonstrating a single hair embedded within the central cornea (white arrow) with surrounding stromal haze.

On examination, her vision was 20/20 bilaterally with intraocular pressures of 17 and 14 mmHg in the right and left eyes, respectively. Slit-lamp exam of the left eye revealed a vertical, black foreign body resembling a human hair within the cornea (Figure 1) with surrounding stromal haze and punctate epithelial erosions. The foreign body was obliquely embedded within the cornea, with the superior aspect displaced more anteriorly just below the epithelium and the posterior aspect within the anterior third of the corneal stroma. The anterior chamber demonstrated moderate inflammation and the remainder of her examination was unremarkable.

Upon further questioning, the patient endorsed a lone incident of a corneal abrasion of the left eye roughly four to five years ago secondary to the edge of a price-tag attached to a pair of sunglasses that she had tried on. She had not experienced any ocular symptoms until her presentation eight weeks prior to her referral to our service.

**Figure 2:**
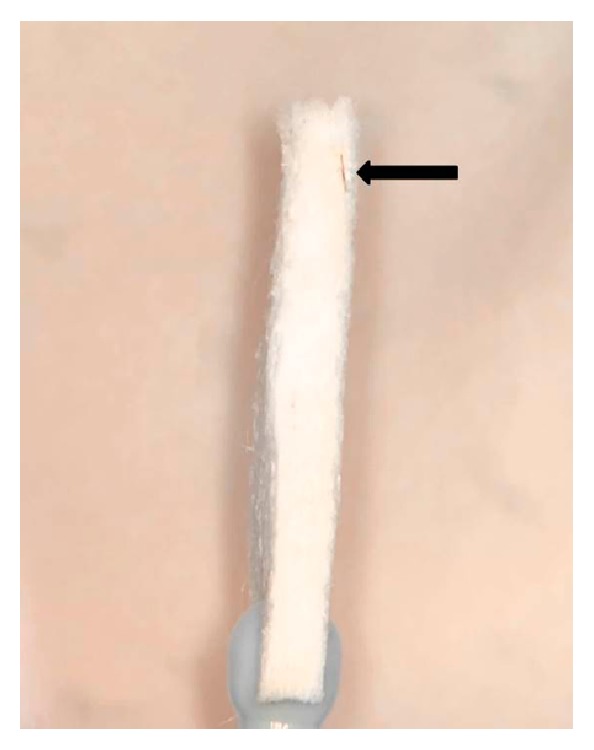
Clinical photo of the hair foreign body (black arrow) on a weck-cell following removal under local anesthetic with a microblade and jeweler's forceps.

The patient was taken to the procedure room where the hair was removed (Figure 2) under local anesthetic using jeweler's forceps, a microblade, and a 30-gauge needle under sterile technique. The remaining edge of loose epithelium was debrided from the wound, a bandage contact lens was placed on the cornea, and the patient was prescribed moxifloxacin drops four times per day for one week. Seven days following removal, the patient had a visual acuity of 20/20 with complete resolution of her anterior chamber inflammation and ocular symptoms.

## 3. Discussion

In the ophthalmic literature, traumatic implantation of hair within the cornea typically involves animal cilia. For instance, McAnena reports a case of an 11-year-old boy with keratitis following trauma from the abdominal hairs of a tarantula [2]. This often results in a localized inflammatory response with a great degree of difficulty removing the hairs due to a barbed design of the cilia. However, reports of human hair embedded within the cornea are sparse. We found only three other cases of human hair within the cornea following trauma [3–5].

It has been reported that eyelashes may migrate into the cornea through clear corneal incisions from surgery [6]. In our case, the patient conceivably had her own human hair follicle embedded following a corneal abrasion with subsequent reepithelialization and no sequelae for over four years. However, the hair was not further analyzed to indisputably verify that it was of human origin.

The consequences of intraocular cilia are variable. There have been reports of uveitis, endophthalmitis, and focal keratitis, whereas others may remain quiescent for years within the stroma [3, 4]. In this case, the authors decided to remove the foreign body due to the patient's symptomatic presentation with persisting irritation, pain, and photophobia despite topical treatment with steroids. Thus, we recommend observation if there is a suspicion that human cilia are embedded within the corneal stroma, unless a focal inflammatory response is elicited with significant comorbidity experienced by the patient. If so, topical steroid therapy is not recommended, as chronic cases of embedded corneal hair may be removed with good visual outcome under local anesthetic.
